# Sensors, Students, and Self: Exploring Knowledge, Self-Efficacy, and Interest Impact of Ocean Sensor Learning on High School Marine Science Students

**DOI:** 10.3390/s22041534

**Published:** 2022-02-16

**Authors:** Travis W. Windleharth, Colin Katagiri

**Affiliations:** Foundry10, Seattle, WA 98105, USA; colin@foundry10.org

**Keywords:** sensors, marine science, ocean science, ocean technology, self-efficacy, STEM interest

## Abstract

This study examined the effect of online technical lessons of how ocean sensors function on student interest in ocean science technology, as well as knowledge gain outcomes. Additionally, the study contributes novel findings to sensor-based learning literature by measuring changes to self-efficacy and confidence gains stemming from sensor-based learning, as well as changes in interest in ocean careers. The area of educational focus was also novel—focusing on how the sensors themselves function, not just what they do. Precipitated by COVID-19 pandemic constraints, the team used a remote learning approach to provide lessons on sensors at a distance, providing an additional opportunity to contrast this approach with previously studied hands-on learning modes. A sample of students from four high school marine science classes completed two assessments both before and after a series of lessons on ocean sensors. This included a self-reported survey (N = 48), and an open-ended knowledge assessment (N = 40). Results showed modest gains in knowledge assessments, and students experienced statistically significant gains in confidence in their ability to explain what sensors are, confidence in their ability to use sensors and understand resulting data, and confidence in accuracy of sensor data (*p* < 0.05). No changes were observed for several measures of interest in ocean technology, nor were there changes in an already high belief that understanding these sensors is important to marine science careers. Notably, these findings measure a positive shift in several measures of self-efficacy and confidence, which is a new finding for sensor-based learning. The findings also contrast with prior related work that included hands-on activities with sensors, which reported an increase in interest after working with sensors, whereas this intervention did not. This suggests a hands-on component is key to increasing interest in ocean technology.

## 1. Introduction

### 1.1. Study Background

The background of this work consists of prior research on student learning with sensors and is situated alongside the body of work exploring the efficacy of teaching marine and environmental science with sensor technology. With the rise of inexpensive microcontrollers such as Raspberry Pi and Arduino, and low-cost environmental sensors that work with these devices, there is increased interest from educators in exploring the potential of this technology in learning settings [1,2]. This interest is an extension of an existing movement towards integrating hands-on instrumentation as part of experiential learning in the classroom [3]. This interest extends to settings of marine and ocean science. As a scientific field, marine and ocean sciences rely on an array of instrumentation to collect many types of data from ocean environments, which frequently use scientific instruments, dependent on various types of sensors. As a result, there has been increased interest in exploring aspects at the intersection of student learning and learning about sensors [4,5].

The most relevant work informing this study is prior work that has been done with sensors in learning environments, measuring several types of outcomes and benefits to working with sensors. For example, Iskander et al. [3] examined the results of 25 sensor-based experiments conducted by the Revitalizing Achievement by Using Instrumentation in Science Education (RAISE) project and concluded that working with sensors in a classroom environment effectively promoted interest in engineering careers and contributed to positive academic gains in testing outcomes [3]. Hotaling & Stolkin [6] also built a case for introducing more sensor-based learning into curricula stating, “to address necessary technology skills, engage underrepresented audiences, and broaden the nation’s technological talent base, all students must be provided with more opportunities to learn practically rather than theoretically—through self-motivated discovery, as opposed to didactic lecturing—and in collaborative learning environments using technology-based problem solving” [6] (p. 40). In addition, Iskander et al. implemented a sensor project with four public high schools as part of the RAISE project [7].

A limited amount of work has been done studying the impact of integrating sensors in the ocean sciences. Seroy et al. (2020) [5] used student-built pH sensors to bridge chemistry and ocean environmental learning and concluded that the hands-on work with the sensors positively impacted student learning. Similarly, Hotaling & Stolkin [6] used a four-module sensor development and deployment program called SENSE-IT (Student Enabled Network of Sensors for the Environment using Innovative Technology) to explore student learning with sensors. This involved using a set of sensors, such as temperature and turbidity sensors, to explore water quality and provide a meaningful learning context. This team found that the lessons had a positive impact on student math scores. On a concerning note, this work also found lower confidence among female participants about their answers compared to male students, even when answers were correct. The RAISE project also included some intervention activities that used sensors important to marine science careers, such as temperature, pH, and dissolved oxygen [7]. Meanwhile, efforts are underway to build and deploy educational programming within the domain of ocean sciences, specifically using ocean sensors. For example, SeaState is a program operated by the University of Washington School of Oceanography, “to dramatically increase K20 STEM knowledge through experiential learning focused on the design, building and implementation of sensors in local marine systems” [8] (p. 1). Boss & Lofton describe the effectiveness of a semester long undergraduate course in the School of Marine Sciences in at the University of Maine. They note, “those who complete this class are not expert programmers, electronic gurus, or roboticists. However, they all have a new appreciation for the role technology plays in oceanography as well as basic literacy in this subject” [9] (p. 221). The authors noted that three students also credited the course with influencing their choice to work in marine technology. Furthermore, Levine et al. 2020 describe the results of as successful project where students created their own bathymetry sensors and deployed them [10].

The subject of online learning has also been addressed in prior work, elements of which are related to this study [11,12,13]. Much of this work compares the efficacy of in-person versus online learning. For example, Anderson aggregated research and perspectives on online learning theory and practice in a seminal compilation [14], and Sadeghi explored the benefits and limitations of distance learning [15]. This prior work established a range of findings about effective methods to conduct online learning. For example, Tzu-Chi studied the effects of online learning and self-regulated learning using a 2 × 2 factorial model [16]. This study found that student learning was positively affected by self-regulated learning supports such as tools to set goals, manage time, strategize, and self-reflect. Chen et al. used a survey method to explore effective design elements for online STEM courses [17]. This team noted that integrated active learning activities and interactive engagement strategies increase effectiveness of STEM learning online. Other researchers have also noted the importance of interactivity in the adoption of learning technologies [18,19]. These frameworks provide a backdrop of the online-only intervention. Several tools consistent with positive outcomes in this body of literature were present in the online lessons provided in the study, including time management tools, designed opportunities for questioning and self-reflection, and interactive engagement strategies during the lessons.

### 1.2. Rationale for Study

Within this body of work on learning with technology, and learning with sensors specifically, there is opportunity to explore the impacts of working with sensors on novel areas. Specifically, measures of self-efficacy tied to sensor-based interventions are absent from most or all prior studies, and the area of pedagogical focus has been on what sensors do, rather than how they work. For the most part, prior interventions on the interface between the scientific instruments and the environment focus on what work the instrument can do, what it measures, and the relevance of these dimensions to the environment. In prior research, less emphasis is placed on the physical and chemical workings of the sensors themselves. In this work, we endeavor to specifically focus on opening the “black box” of how the sensors at the heart of instruments work. For example, examining in detail how stress gauge transducers bend with changes in pressure, which affects their electrical resistance and, therefore, signal strength.

Additionally, due to the COVID-19 pandemic, this work differs from most other scholarship on student learning about sensors, as most interventions in the literature include a hands-on component where students work directly with sensors, by building or deploying them. Due to pandemic constraints on working with youth, these conditions presented an opportunity to test an intervention approach that consisted solely of presentations on the deeper technical details of how sensors work, instead of using a hands-on approach. No hands-on component was included.

This work builds on the limited existing research exploring the relationship between learning about sensors, and other education outcomes. Specifically, the team is probing understanding of the sensor learning intervention in one or more of several areas (i). student confidence in instrumentation and the findings from their data, (ii). student self-efficacy, and (iii). student engagement and interest. Student confidence and self-efficacy is of interest in career and STEM learning research. For example, social cognitive career theory (SCCT) connects learning experiences to increases in self efficacy, leading to career goals. (Lent et al., 2002), and prior work has connected self-efficacy to successful STEM outcomes (Rittmayer et al.) [20,21]. As such, interventions that increase student self-efficacy with domains or concepts are important experiences for learners.

### 1.3. Research Questions

This work seeks to answer the following three research questions:

RQ1. To what extent can short lessons on sensors increase high school student understanding of what they do and how they work?

RQ2. Does learning how ocean sensors function affect student sense of self efficacy and confidence?

RQ3: Do traditional lectures on sensor technology increase student interest in ocean careers and ocean technology?

There are three original elements to this intervention, and each leads to a specific implication for the field of sensor-based learning. First, there are implications to the design of sensor based learning activities, stemming specifically from the strong emphasis in the instruction on the physical and chemical mechanisms that cause sensors to work (R1). Outcomes from this intervention will help inform the extent to which unlocking the “black box” of what makes sensors work may impact outcomes of these programs. Second, there are implications stemming from the self-efficacy measures, which is an original dimension this study explores (R2). Building on the growing body of research linking student self-efficacy and positive learning with career outcomes, there are implications to understanding the extent to which learning the technical details of how sensors work impacts student self-efficacy. As more educational institutions look to low-cost microcontrollers and sensor-based activities, understanding the extent to which learning how these technical details can affect self-efficacy has implications for the design and even appropriateness of use of future sensor-based learning programs. Third, there are implications of learning what differences in some measures (notably engagement and interest) differ from prior work by focusing more deeply on sensor functions, in lieu of a hands-on component (R3). Most prior work on sensor-based learning in classrooms involves hands-on activities, and circumstances caused by the COVID-19 pandemic provide an opportunity to detect whether measures of interest and engagement differ if lessons are delivered remotely, and do not include a hands-on component. This has implications for the consideration of modes of instruction as educators and informal learning institutions develop new sensor-based interventions to take advantage of this increasingly important and accessible technology.

## 2. Materials and Methods

This fundamental intervention in this study consisted of presenting a series of lessons on the precise physical and chemical mechanisms that six types of ocean sensors use to function to a sample student population. The research questions were addressed by collecting both pre-test and post-test data from the same sample. The sole intervention for this study was the presentation of a series of technical lessons on sensor function, with the main goals of (1) ensuring that learners understand what a sensor is, and (2) ensuring learners gain a concrete understanding of how each of the six sensors worked at a fundamental level. These lessons were presented in series and explicitly walked participants through technical information about the function of each sensor. Data were collected using two instruments, including an open-ended knowledge assessment about what participants believed sensors did, and how they believed they functioned, as well as a self-report Likert scale survey addressing the self-efficacy and interest questions. The two instruments were administered before the sensors and learning activity, as well as shortly afterward to explore any impact the intervention had on the measures by noting changes between the pre- and post-tests.

The sample for this study was a convenience sample consisting of students in four high school classes on marine science, all located in public schools in the Puget Sound area of Washington State. All students within the four classes were invited to participate in the study, and a total of eighty-six students participated in the class sessions on the ocean sensors. Each class was a dedicated Marine Science course of high school students. Most students in these classes and sample were 11th and 12th graders; however, a small subset of the population were 10th graders eligible to take the course as they were on a “highly capable” academic track. This convenience sample was chosen as the authors had pre-established working relationships with these learning institutions, particularly during the COVID-19 pandemic when externally directed led interventions by the team were not possible.

The focus of the instruction was a set of six ocean sensors that are in operation by marine science researchers. Table 1 below lists the specific sensors covered in the lesson, and the ocean properties they measure. The area of focus in each case is the specific sensor that interacts with the ocean environment to generate an electrical signal that is transformed into a signal reading, as opposed to the overall purpose of the instrument more generally. This means looking at the specific interface of a sensor object with the environment, and how the mechanical, chemical, and electrical processes involved reflect properties of the environment through the signals they generate. With this focus, researchers sought to focus acutely on the sensors themselves, and describe sensor function in concrete terms. The research team delivered the lesson content to classes virtually over a teleconference with each of the four classes, each in a separate session. The run-time for all lessons ranged from 45 to 55 min. Students were also given 15 to 20 min to ask questions and collect clarifying information.

Two instruments were used to collect data. The first was a self-assessment, consisting of a series of ten questions organized into three categories, self-efficacy, trust in data, and domain interest. A ten-point scale was used to account for a potential ceiling effect, as the students in the classes included in the research self-selected these optional courses of study due to at least some interest in the field. This instrument was designed to measure the abstract self-reported values in the study. The questions on this assessment appear below in Table 2. For both the survey and the knowledge assessment, only student responses that included both a complete pre-test and post-test were included in the final data set from each instrument. After data cleaning and review under these criteria, the final data set included a somewhat lower number of self-reported survey responses (N = 48), compared to knowledge responses (N = 40).

The open response instrument repeats a set of questions for each instrument. First, “Do you know what a/an [sensor] is?”, which includes the fixed responses “Yes”, “No”, I think so”. Additionally, a second question for each instrument asks, “how do you think a/an [sensor] works?” For this question, students were provided unlimited space for an open response. A rubric was created for the purpose of scoring student open responses. The scoring rubric for open response question, including examples, is presented in Appendix A as Table A1.

## 3. Results

### 3.1. Results for Student Self-Report

Results of the student self-reported items are reported below as Table 2. Per the method described, this reflects each item as rated on a ten-point Likert scale. The first four items were found to have statistically significant changes. Notably, the sixth item, on interest in ocean technology, was close to significant (*p* = 0.066) but was not within significance threshold.

### 3.2. Student Knowledge Assessment Results

#### 3.2.1. Student Awareness of Sensors

This section presents the results of the knowledge response instrument assessing student understanding of what oceans sensors did, and how they work. The first part of these results aggregate student responses to the question “Do you know what a/an [sensor] is?” This question was repeated for each sensor. Table 3 below shows the responses for the ADCP, pH, and DO sensors, and Table 4 presents this information for the three sensors on the CTD instrument (conductivity, temperature, depth). These tables show the pre- and post-test responses side by side.

#### 3.2.2. Student Sensor Knowledge, Scored Open Responses

This section presents the results of the knowledge response instrument assessing student understanding of sensors. These were scored with the rubric ranging from 0 to 3 points. A peculiarity occurred with this instrument, with many students’ open responses in both the pre-test and post-test straying from discussing the function of the sensor and repeating the function of the instrument as opposed to physical or chemical principles related to the sensor itself. For example, “it tests the water’s pH” for the pH sensor, or “it sends out sound waves” for the ADCP. The pre-established rubric necessitated scoring responses that did not address the sensor “0”. The results for the ADCP, pH, and DO sensors are listed below in Table 5, and the conductivity, temperature, and depth sensor scores are listed below in Table 6.

Notably, while many post assessment responses remained at 0 or did not change, no open assessment score among 40 students decreased from pre-test to post-test for any of the six sensors discussed. The increase in student scores from pre-test to post test is listed below in Table 7.

## 4. Discussion

With regard to R1: “To what extent can short lessons on sensors increase high school student understanding of what they do and how they work?”, the data show positive gains in this area. Across the board, student responses to the question as to whether they knew what a particular sensor was increased. These results show modest overall improvements among student open responses after the intervention. Notably, student scores dropped from the pre-test to the post test; however, many responses were rated at “0” in both the pre-test and post-test, largely due to tautological responses about the role or function of the instrument, as opposed to the function of the sensor. Despite this, many students improved their scores, demonstrating a modest knowledge increase about the sensors in question.

With the student self-assessment, of the five questions addressing self-efficacy and confidence, four resulted in statistically significant gains. These address R2: “Does learning how ocean sensors function affect student sense of self efficacy and confidence?” and affirm gains in this area. Among all questions, student beliefs that they can explain what a sensor is increased the most, shifting from an average of 4.91 before the intervention to 7.15 afterward, a gain of 2.24 points. Additionally, student confidence in the accuracy of data generated by ocean science sensors increased by 0.74 points, student confidence in their ability to use ocean science sensors increased by 1.78 points, and student confidence in their ability to understand data from the sensors increased 1.76 points. The only question in the self-efficacy cluster that saw no statistically significant shift was student belief that they can learn the technical details of how sensors work. In this case, students were already reasonably confident that they could learn this, with a pre-test score of 6.96. The intervention appears to have had a meaningful positive impact on several aspects of student self-efficacy.

These results suggest that learning about the specific way ocean sensors operate can increase aspects of student self-efficacy around understanding and using sensors, as well as confidence in data, and appear to be novel findings. Importantly, this represents one type of intervention (in-depth technical lessons delivered online), and further work is needed to see if gains in these measures hold in other delivery modes (such as in-class, hands on learning).

There were no statistically significant changes in student scores among any of the five questions pertaining to interest in and the importance of understanding ocean technology. Thus, the question R3: “Do traditional lectures on sensor technology increase student interest in ocean careers and ocean technology?” appears to be no for this particular style of online and in-depth technical lesson. The question “how interested are you in ocean science technology?” dropped after the intervention by 0.46 points; however, this change was not statistically significant. There was also no change in student interest in the way ocean sensors work, nor was there a change in student expectation that they would spend extra time learning about an ocean sensor when using a new instrument.

There were also no meaningful changes to student beliefs about the importance of understanding sensors to understand ocean related knowledge, nor were there changes to the belief that understanding sensors was important to ocean science careers. However, these two questions had the highest average student responses in the pre-survey, so it appears there is a ceiling effect that could be occurring with these responses. In the pre-test, the average student response relating the importance of sensor knowledge to ocean knowledge was 7.74, and student belief in the importance of “understanding technical details of how ocean sensors work in ocean science careers” recorded the highest response of the study, at 8.41. Students appeared to already believe that understanding the specific technical workings of ocean sensors increases their ability to understand ocean knowledge and would be important to an ocean science career.

The findings in this study that indicate student interest in ocean technology, student interest in learning how learning how sensors work, and student expectation of taking extra time to learn about sensors when introduced to new instruments in the future did not change, and is a contrasting result with what was found in related prior work. For example, Iskander et al., Seroy, et al., and Hotaling, all found an increase in student interest toward some aspect of sensor or technology after their intervention [3,5,6]. This study cannot provide a clear answer as to why this was the case; however, the design raises some possibilities. One major difference between each of these prior studies and this study (and perhaps the most significant difference) is that this study did not include any direct interaction by students with actual sensors, while the above cited work did. A second major difference is that the emphasis in instruction was on the detailed technical workings of the sensors. All instruction and interaction in this study were based on slide presentations and lectures delivered remotely via the teleconference software Zoom, and the intervention included no hands-on component. Limitations of the COVID-19 pandemic on in-person interaction in schools prevented the team from deploying hands-on activities with sensors. One possibility is that this could indicate that a hands-on component of learning with sensors is a critical element to positively impacting student interest in sensors and related technology. This notion is supported by other prior work such as Sobhan, et al., Mahaffey et al., and Walia et al. [22,23,24]. Boss & Loftin also make an explicit argument for hands-on learning with sensors and argue that students experiencing hands-on learning with ocean sensors gained self-confidence and greater appreciation of the subject [9]. To test this question specifically, a future study could use the same fundamental lesson plan, but also include a hands-on component.

In summary, this study allows us to conclude that lesson-only interventions teaching students about the specific technical workings of sensors does improve student understanding of sensor function. This study also demonstrates for the first time that this type of intervention positively impacts some dimensions of student self-efficacy and confidence. However, this study differs from prior work in that no increase in student interest in ocean science technology or instrumentation was detected. Notably, student beliefs that understanding ocean sensor function is important were already high before the intervention and did not change because of the lessons.

In a future study, the team intends to replicate this work using the same method, and same sensor learning materials, while also including a hands-on component for each of the sensors of interest. Results from this or other follow up studies could further test the importance of directly working with technology in increasing student interest and engagement, and test whether student knowledge of sensors improves even more when lecture lessons are coupled with hands-on learning. This future iteration could also see if positive shifts in student self-efficacy and confidence also hold when a hands-on component is included in the intervention.

## Figures and Tables

**Table 1:** Summary of instrument sensors covered in intervention lessons.

Instrument	Function	Sensor	Measure
Teledyne Acoustic Doppler Current Profiler (ADCP)	Measures current speed	Strain Gauge Transducer	Velocity (m/s)
Sea-Bird SeapHOx CTD Sensor Suite	Measure water conductivity	Ammeter	Current (S/m)
	Measure water temperature	Thermistor	Temperature (C)
	Measure depth from surface	Strain gauge transducer	Pressure (decibar)
Sea-Bird SeapHOx pH Sensor	Measure acidity of water	Ion Sensitive Field Effect Transistor (ISFET)	Acidity (pH)
Sea-Bird SeapHOx DO Sensor	Measures dissolved oxygen	Strain Gauge Transducer	Ml/L dissolved oxygen

**Table 2:** Pre- and post-test self-assessment results.

Question	Before Mean	After Mean	Change	Significance
How confident are you that you can explain what a research sensor is?	4.91	7.15	2.24	*p* < 0.0001
How confident do you feel in the accuracy of data generated by ocean science sensors?	7.39	8.13	0.74	*p* = 0.007
How confident do you feel in your ability to correctly use ocean science sensors?	4.04	5.83	1.78	*p* < 0.0001
How confident do you feel in your ability to understand data generated from ocean science sensors?	5.35	6.80	1.46	*p* < 0.0001
How confident are you that you can learn the technical details of how ocean sensors work?	6.96	7.15	0.20	*p* > 0.05
How interested are you in ocean science technology?	7.00	6.54	−0.46	*p* = 0.05
How interested are you in the details of how ocean instrument sensors work?	6.33	6.07	−0.26	*p* > 0.05
How likely are you to spend extra time learning about how the sensors work when using a new ocean instrument?	5.30	5.33	0.02	*p* > 0.05
How much do you think understanding how ocean sensors work increases one’s ability to understand related ocean knowledge?	7.74	7.50	−0.24	*p* > 0.05
How important do you think it is to understand technical details of how ocean sensors work in ocean science careers?	8.41	8.11	−0.30	*p* > 0.05

**Table 3:** Student knowledge of what sensor is (ADCP, pH, and DO).

Response	ADCP before	ADCP after	pH before	pH after	DO before	DO after
Yes	10	17	21	30	11	26
No	27	11	5	1	14	5
I think so	10	11	12	7	14	7

**Table 4:** Student knowledge of what a sensor is (CTD).

Response	Conductivity before	Conductivity after	Temperature before	Temperature after	Depth before	Depth after
Yes	9	15	30	30	15	24
No	25	7	2	1	6	4
I think so	5	17	7	8	18	11

**Table 5:** Student scored open response assessment (ADCP, pH, and DO).

Response	ADCP before	ADCP after	pH before	pH after	DO before	DO after
0	23	18	33	23	37	28
1	10	8	4	8	2	2
2	5	12	2	4	0	5
3	1	1	0	3	0	4

**Table 6:** Student scored open response assessment (CTD).

Response	Conductivity before	Conductivity after	Temperature before	Temperature after	Depth before	Depth after
0	27	21	33	28	37	31
1	8	2	4	3	1	2
2	2	6	0	1	0	0
3	0	10	1	7	1	6

**Table 7:** Number of open response student scores that improved before and after intervention by sensor type.

Sensor	Number of Students Improved	% Increase
ADCP	13	33%
pH	21	53%
DO	11	28%
Conductivity	15	38%
Temperature	10	25%
Depth	7	18%

## Data Availability

Data from this study was collected by the team for the purpose of this study, and is stored on internal servers within foundry10.

## Appendix A. Scoring Rubric

**Table A1:** Scoring Rubric for Open Response Questions.

Score	Criteria	Examples	Reasoning
0	Non-response, or completely inaccurate	(Blank), “I don’t know”, “A Dumb Cruel Pig”	data
1	Educated guesses, or contains one correct fact, but other information is missing or wrong and there is no discussion of the sensor	(A) “Possibly a location finder or something that involves sound waves” (B) “Sends sound waves to find surfaces”	Includes one key fact—sending sound waves—but otherwise is incorrect, and there is no mention of the sensor itself
2	Information that at least one piece that is accurate, and one piece that is inaccurate, or functional characteristics are correct, but answer does not address the sensor details	(A) “It sends out short pings at inaudibly high frequencies and then measures their distortion and “flight time” as they bounce off the water particles around them” (B) “Uses the doppler effect to measure the distance of things, pitch changes based on what direction said thing is moving”	(A) This answer has the “functional” piece, but does not talk about the sensors (e.g., transducer), 3. (B) Also correct, and has most of the info, but still no mention of transducer or resistance change or any other clue that they understand the sensor itself
3	Includes both systemic and sensor components, with no fundamental inaccuracies	(A) “Measures depth by using a metal filament (transducer), which warps and deforms with pressure and effects conductivity, which can be recorded”, (B) “Light is emitted into an oxygen molecule, and a photodiode measures the decay of fluorescence due to oxygen content, which is inversely proportional to light”	These responses accurately describe how the sensor itself works
